# Genomic and proteomic profiling II: Comparative assessment of gene expression profiles in leiomyomas, keloids, and surgically-induced scars

**DOI:** 10.1186/1477-7827-5-35

**Published:** 2007-08-24

**Authors:** Xiaoping Luo, Qun Pan, Li Liu, Nasser Chegini

**Affiliations:** 1 Department of Obstetrics and Gynecology, University of Florida, College of Medicine, Gainesville, Florida 32610, USA; 2 Interdisciplinary Center for Biotechnology Research, University of Florida, College of Medicine, Gainesville, Florida 32610, USA

## Abstract

**Background:**

Leiomyoma have often been compared to keloids because of their fibrotic characteristic and higher rate of occurrence among African Americans as compared to other ethnic groups. To evaluate such a correlation at molecular level this study comparatively analyzed leiomyomas with keloids, surgical scars and peritoneal adhesions to identify genes that are either commonly and/or individually distinguish these fibrotic disorders despite differences in the nature of their development and growth.

**Methods:**

Microarray gene expression profiling and realtime PCR.

**Results:**

The analysis identified 3 to 12% of the genes on the arrays as differentially expressed among these tissues based on P ranking at greater than or equal to 0.005 followed by 2-fold cutoff change selection. Of these genes about 400 genes were identified as differentially expressed in leiomyomas as compared to keloids/incisional scars, and 85 genes as compared to peritoneal adhesions (greater than or equal to 0.01). Functional analysis indicated that the majority of these genes serve as regulators of cell growth (cell cycle/apoptosis), tissue turnover, transcription factors and signal transduction. Of these genes the expression of E2F1, RUNX3, EGR3, TBPIP, ECM-2, ESM1, THBS1, GAS1, ADAM17, CST6, FBLN5, and COL18A was confirmed in these tissues using quantitative realtime PCR based on low-density arrays.

**Conclusion:**

the results indicated that the molecular feature of leiomyomas is comparable but may be under different tissue-specific regulatory control to those of keloids and differ at the levels rather than tissue-specific expression of selected number of genes functionally regulating cell growth and apoptosis, inflammation, angiogenesis and tissue turnover.

## Background

Leiomyomas are benign uterine tumors with unknown etiology that originate from transformation of myometrial smooth muscle cells and/or connective tissue fibroblasts during the reproductive years. Leiomyomas can develop in multiple numbers that are individually encapsulated by a connective tissue core separating them from the surrounding normal myometrium and are ovarian steroid-dependent for their growth. Although they occur independent of ethnicity, clinical and epidemiological studies have indicated that African Americans are at a higher risk of developing leiomyomas compared to other ethnic groups [1].

Leiomyomas have also often been compared to keloids because of a higher rate of occurrence in African Americans and their fibrotic characteristics despite differences in the nature of their development and growth [2]. Keloids are benign skin lesions that develop spontaneously, or form from proliferation of dermal cells following tissue injury resulting in a collagenous and poorly vascularized structure at later stage of their development [3-6]. Unlike surgically-induced and hypertrophic scars that are confined to the area of original tissue injury, keloids can expand beyond the boundaries of their original sites following removal and during healing. Keloids are rather similar to hypertrophic scars at early stages of development, however they become collagenous and poorly vascularized at later stages and tend to occur more frequently in darker skinned individuals [3,4]. Surgically-induced injury and/or inflammation also result in peritoneal scar or adhesions and similar to other incisional scars they are confined to the area of tissue injury[7]. Peritoneal adhesions also display a considerable histological similarity with dermal scars; however there is no data to suggest a higher risk of adhesion formation with ethnicity. Comparatively, uterine tissue injury i.e., following myomectomy or cesarean sections, does not cause leiomyomas formation, but rather results in incisional scar formation at the site of injury. Furthermore, leiomyomas consist mainly of smooth muscle cells forming a relatively vascuraized tissue, while keloids derive from proliferation of connective tissue fibroblasts, adopting a myofibroblastic phenotype at a later stage of wound healing[3,4].

As part of these characteristics previous studies have identified excess production and deposition of extracellular matrix, namely collagens in leiomyomas, keloids, hypertrophic and surgical scars and peritoneal adhesions [2,7-10]. Evidence also exists implicating altered production of several proinflammatory and profibrotic cytokines, proteases and adhesion molecules in pathogenesis and characteristic of these and other fibrotic disorders [11-14]. Large-scale gene expression studies have provided additional evidence for the expression of a number of differentially expressed genes in leiomyomas [11,15-17], keloids and hypertrophic scars [15,16] as compared to their respective normal tissues. Several conventional studies have demonstrated that the products of some of these genes regulate various cellular activities implicated in the outcome of tissue fibrosis at various sites throughout the body Among these genes, include several growth factors and cytokines such as TGF-β system, proteases, adhesion molecules and extracellular matrix etc. (for review see [7-17]). Despite these advancements, the biological significance of many of these genes in pathophysiology of leiomyomas and keloids and their relationship to the outcome of other tissue fibrosis remains to be established. In addition, there has not been any study that comparatively analyzed the molecular profile that distinguishes leiomyomas from other fibrotic tissues, specifically keloids.

Considering these characteristics we used large-scale gene expression profiling to evaluate such a correlation at molecular level by comparatively analyzing leiomyomas with keloids, surgical scars and peritoneal adhesions to identify genes that are either commonly and/or individually distinguish these fibrotic disorders despite differences in nature of their development and growth. We evaluated the expression of 12 genes in these tissues representing several functional categories important to tissue fibrosis using quantitative realtime PCR based on low-density arrays.

## Methods

All the materials and methods utilized in this study are identical to our previous studies and those reported in the accompanying manuscript [11,17]. Prior approval was obtained from the University of Florida Institutional Review Board for the experimental protocol of this study, with patients with scars giving informed consent, while the study with leiomyomas was expedited and did require obtaining written informed consent.

Total cellular RNA was isolated from keloid/incisional scars (N = 4) and subjected to microarray analysis using human U133A Affymetrix GeneChips as described in the accompanying manuscript [17]. One patient who had developed keloid at the site of previous surgical incision also developed leiomyoma. All the patients with keloids and one patient with incisional scar were African Americans. In addition, we utilized the gene expression data obtained from our previous study [11] involving leiomyomas (N = 3) and peritoneal adhesions (N = 3) using human U95A GeneChips. These tissues were from Caucasians patients with the exception of one peritoneal adhesion collected from an African American patient. The age of patients with leiomyomas ranged from 29 to 38 years. These women were not taking any medication, including hormonal therapy, for pervious 3 months prior to surgery and based on their last menstrual period and endometrial histology was from early-mid secretary phase of the menstrual cycle. The age of patients with adhesions ranged from 25 to 46 years and those with keloids and surgical scars were 26, 32 and 39 years, respectively. All the tissues with the exception of one keloid matched by their corresponding normal tissues i.e. myometrium, skin and parietal peritoneum for microarry analysis. All the procedures for total RNA isolation, amplification, cDNA synthesis, RNA labeling and hybridization into the GeneChips were carried out as previously described in detail [11].

### Microarray data analysis

The gene expression values obtained from the leiomyomas and matched myometrium (N = 6) using U133A GeneChips in the accompanying manuscript was utilized here only for the purpose of comparative analysis. The gene expression values obtained from all U133A and U95A GeneChips were independently subjected to global normalization and transformation, and their coefficient of variation was calculated for each probe set across the chips as previously described [11]. The selected gene expression values were than subjected to supervised learning including statistical analysis in R programming and ANOVA with Turkey test and gene ranking at P ≤ 0.005 followed by 2-fold change cutoff[11]. Functional annotation and molecular pathway analysis was carried out as described [17].

For combining the data from the U95A and U133A chips the probes that were absent across all chips were removed and subjected to t-test to identify differentially expressed genes. The data set was annotated using Entrez Gene and full annotation files NetAffy software and probe sets were consolidated based on Entrez Gene ID and subjected to microarray.dog.MetaAnalysisTester. The analysis keeps one probe for each gene with the smallest p-value for up or down t-test. The probe with smallest p-value for up regulated genes may be different from probe sets with smallest p-value for down-regulated genes. When the data from U95A and U133A was combined if a gene was represented on one platform, but not on both the missing data was replaced with NA. The data was subjected to Fisher combine p-values using inverse chi-square method and permutation test to determine new p-value, named randomized inverse chi-square p-value and to calculate the traditional inverse chi-square p-value. The false discovery rate was calculated using the inverse chi-square p-value and the min t-test p-value for each gene.

### Quantitative realtime PCR

The same total RNA isolated from these tissues and used for microarray studies was also subjected to quantitative realtime PCR using custom-made TaqMan Low Density Arrays (LDAs) assessing the expression of 12 genes and the house-keeping gene, GAPDH. Detailed descriptions of LDA and realtime PCR, including data analysis has been provided in the accompanied manuscript[17].

## Results

### Gene expression profiles of leiomyomas, keloids and scars

**Figure 1:**
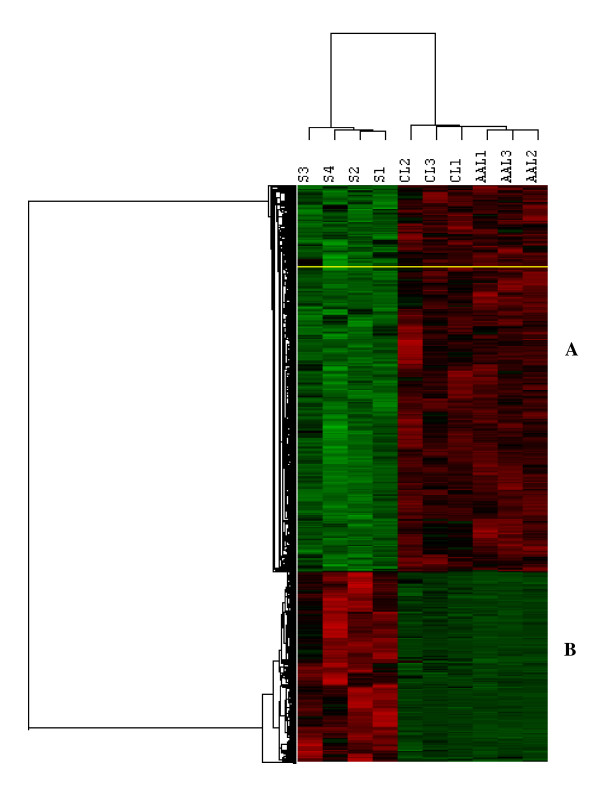
Cluster analysis of 1124 differentially expressed transcripts in leiomyomas (N = 6) form African Americans (AAL1, AAL2 and AAL3), Caucasians (CL1, CL2, and CL3) and in keloids (S3 and S4) and incisional scars (S1 and S2) identified following supervised and unsupervised analysis and p ranking of P < 0.005 followed by 2-fold cutoff change selection (Affymetrix U133A). Genes represented by rows were clustered according to their similarities in expression patterns for each tissue identified as A and B. The dendrogram displaying similarity of gene expression among the cohorts is shown on top of the image, and relatedness of the arrays is denoted by distance to the node linking the arrays. The incisional scar (S1) and keloids were from African American patients. The shade of red and green indicates up- or down-regulation of a given gene according to the color scheme shown below.

Utilizing Affymetrix U133A platform we first assessed the gene expression profile of keloids and incisional scars. Following supervised and unsupervised assessments of the gene expression values in each cohort the combined data set with the gene expression values of leiomyomas reported in the accompanying manuscript using U133A arrays [17] only for the purpose of comparative analysis. The analysis based on supervised and unsupervised assessment and P ranking of P < 0.005, followed by 2-fold cutoff change selection, resulted in identification of 1124 transcripts (1103 genes) of which 732 genes were over-expressed and 371 were under-expressed in leiomyomas as compared to keloids/incisional scars (N = 4). Hierarchical clustering separated these genes into distinctive groups with each cohort clustering into the corresponding subgroup (Fig. 1). A partial list of these differentially expressed genes with their biological functions is shown in Tables 1 and 2. The combined gene list presented in Tables 1 and 2 is different from the list reported in the accompanying manuscript for leiomyomas[17], although many commonly expressed genes displaying different expression values could be find in between the tables.

**Table 1:** List of over-expressed in leiomyomas as compared to scar tissues (keloids/incesional scars)

**Gene Bank**	**Symbol**	**Fold Change**	**Probability**	**Function**
NM_003478	CUL5	5.06	0.0001	apoptosis
AB037736	CASP8AP2	4.07	0.0021	apoptosis
NM_018947	CYCS	2.08	0.0013	apoptosis
AB014517	CUL3	2.07	0.00001	apoptosis
BC010958	CCND2	5.62	0.0041	cell cycle
U47413	CCNG1	3.16	0.0007	cell cycle
AF048731	CCNT2	2.83	0.0004	cell cycle
NM_001927	DBS	61.51	0.0022	cytoskeleton/motility
AK124338	ACTG2	30.16	0.00001	cytoskeleton/motility
BC022015	CNN1	27.26	0.00001	cytoskeleton/motility
NM_006449	CDC42EP3	25.29	0.0051	cytoskeleton/motility
AB023209	KIAA0992	17.61	0.0004	cytoskeleton/motility
AF474156	TPM1	14.84	0.0029	cytoskeleton/motility
BC011776	TPM2	12.04	0.00001	cytoskeleton/motility
M11315	COL4A1	11.87	0.0029	cytoskeleton/motility
AK126474	LMOD1	9.49	0.00001	cytoskeleton/motility
AB062484	CALD1	9.22	0.0042	cytoskeleton/motility
NM_003186	TAGLN	6.68	0.00001	cytoskeleton/motility
BC017554	ACTA2	5.18	0.00001	cytoskeleton/motility
AK074048	FLNA	5.08	0.00001	cytoskeleton/motility
NM_016274	CKIP-1	4.44	0.002	cytoskeleton/motility
BC003576	ACTN1	4.23	0.0024	cytoskeleton/motility
AF089841	FLNC	3.43	0.0005	cytoskeleton/motility
X05610	COL4A2	7.86	0.0017	extracellular matrix
BC005159	COL6A1	3.70	0.002	extracellular matrix
A98730	CAPN6	13.7	0.0023	protease activity
U41766	ADAM9	4.76	0.0021	protease
NM_001110	ADAM10	3.2	0.00001	protease
AF031385	CYR61 (CCN1)	9.13	0.0035	growth factor
M32977	VEGF	7.13	0.002	growth factor
AF035287	SDFR1	4.70	0.0001	chemokine receptor
X04434	IGF1R	3.64	0.0017	growth factor receptor
AB029156	HDGFRP3	2.89	0.0006	GF receptor activity
AF056979	IFNGR1	2.72	0.0001	signal transduction
AB020673	MYH11	53.80	0.0006	signal transduction
D26070	ITPR1	26.18	0.0034	signal transduction
AB037717	SORBS1	15.25	0.0005	signal transduction
AF110225	ITGB1BP2	14.18	0.0009	signal transduction
AB004903	SOCS2	11.39	0.0002	signal transduction
B011147	GREB1	11.37	0.0025	signal transduction
AB000509	TRAF5	7.83	0.0032	signal transduction
NM_005261	GEM	7.48	0.0003	signal transduction
AF028832	HSPCA	4.27	0.00001	signal transduction
AC006581	M6PR	3.85	0.0012	signal transduction
AF275719	HSPCB	3.74	0.001	signal transduction
AJ242780	ITPKB	3.68	0.00001	signal transduction
AK095866	GPR125	3.62	0.0001	signal transduction
AF016050	NRP1	3.44	0.0011	signal transduction
AB015706	IL6ST	3.42	0.0002	signal transduction
AK057120	HMGB1	3.16	0.0001	signal transduction
NM_006644	HSPH1	3.14	0.002	signal transduction
AB072923	BSG	2.90	0.0024	signal transduction
AB010881	FZD7	2.62	0.0024	signal transduction
AF273055	INPP5A	2.58	0.002	signal transduction
AC078943	TANK	2.32	0.0005	signal transduction
AF051344	LTBP4	2.20	0.0002	signal transduction
AJ404847	ILK	4.74	0.0002	protein kinase activity
AF119911	CSNK1A1	3.40	0.0015	protein kinase activity
NM_002037	FYN	3.30	0.0028	protein kinase activity
AB058694	CDC2L5	2.37	0.0001	protein kinase activity
AF415177	CAMK2G	2.18	0.0008	protein kinase activity
NM_005654	NR2F1	12.57	0.0039	transcription factor
BC062602	PNN	9.93	0.0001	transcription factor
AK098174	MEIS1	9.61	0.00001	transcription factor
NM_000125	ESR1	9.36	0.0004	transcription factor
AF249273	BCLAF1	8.62	0.0001	transcription factor
AF017418	MEIS2	7.46	0.0009	transcription factor
AF045447	MADH4	6.39	0.00001	transcription factor
AF162704	AR	5.54	0.0018	transcription factor
NM_001527	HDAC2	4.76	0.00001	transcription factor
NM_004268	CRSP6	4.76	0.0001	transcription factor
BC020868	STAT5B	4.57	0.0003	transcription factor
BC002646	JUN	3.84	0.0042	transcription factor
AY347527	CREB1	3.77	0.0031	transcription factor
AL833643	MAX	3.66	0.0014	transcription factor
NM_021809	TGIF2	3.58	0.0014	transcription factor
AB007836	TGFB1I1	3.55	0.0007	transcription coactivator
NM_005760	CEBPZ	3.53	0.00001	transcription factor
AL833268	MEF2C	3.49	0.0019	transcription factor
NM_005903	MADH5	3.10	0.0037	transcription factor
NM_022739	SMURF2	2.58	0.0013	transcription factor
NM_003472	DEK	2.55	0.0001	transcription factor
NM_001358	DHX15	2.49	0.0029	transcription factor
BC029619	ATF1	2.41	0.0026	transcription factor
AB082525	TSC22	2.26	0.0002	transcription factor
AL831995	MEF2A	2.25	0.0024	transcription factor
AA765457	DDX17	10.41	0.0035	translation factor
NM_018951	HOXA10	8.69	0.00001	translation factor
BC000751	EIF5A	4.07	0.001	translation factor
AF015812	DDX5	2.48	0.0004	translation factor
AL079283	EIF1A	2.35	0.0005	translation factor
NM_003760	EIF4G3	2.35	0.0028	translation factor
NM_012218	ILF3	2.29	0.0003	translation factor
AB018284	EIF5B	2.26	0.002	translation factor
AF155908	HSPB7	9.52	0.0002	protein binding
AF209712	MCP	6.54	0.00001	complement activation
AL833430	SPARCL1	5.12	0.00001	calcium ion binding
AF297048	PTGIS	4.26	0.0004	catalytic activity
AF288537	FSTL1	4.11	0.001	calcium ion binding
AB034951	HSPA8	3.13	0.001	protein binding
NM_001155	ANXA6	2.85	0.0014	calcium ion binding
NM_003642	HAT1	2.81	0.00001	catalytic activity
NM_002267	KPNA3	2.55	0.0031	protein transporter
AK124769	XPO1	2.46	0.0002	protein transporter
AJ238248	CENTB2	2.37	0.0045	GTPase activator activity
AF072928	MTMR6	2.17	0.002	phosphatase activity

Partial list of differentially expressed genes identified in leiomyomas (African Americans and Caucasians) as compared to keloid/incisional scars as shown in Fig. 1. The genes were selected based on p ranking of p ≤ 0.005 and 2-fold cutoff change selection (F. Change) as described in materials and methods. Table 1 displays the over-expressed genes in leiomyomas as compared to keloid/incisional scars.

**Table 2:** List of under-expressed in leiomyomas as compared to scar tissues (keloids/incesional scars)

**Gene Bank**	**Symbol**	**Fold Change**	**Probability**	**Function**
AF004709	MAPK13	0.06	0.0002	apoptosis
AF010316	PTGES	0.09	0.0003	apoptosis
NM_014430	CIDEB	0.21	0.0014	apoptosis
AJ307882	TRADD	0.26	0.0007	apoptosis
BC041689	CASP1	0.31	0.0009	apoptosis
NM_014922	NALP1	0.31	0.0025	apoptosis
AF159615	FRAG1	0.33	0.0044	apoptosis
BC019307	BCL2L1	0.42	0.0027	apoptosis
NM_016426	GTSE1	0.43	0.0033	apoptosis
AK027080	LTBR	0.50	0.0047	apoptosis
M92287	CCND3	0.48	0.0028	cell cycle
AJ242501	MAP7	0.2	0.0001	structural molecule
AF381029	LMNA	0.3	0.00001	structural molecule
X83929	DSC3	0.009	0.0035	cell adhesion
AB025105	CDH1	0.01	0.0009	cell adhesion
AJ246000	SELL	0.21	0.002	cell adhesion
NM_003568	ANXA9	0.22	0.0031	cell adhesion
AF281287	PECAM1	0.36	0.0017	cell adhesion
J00124	KRT14	0.0001	0.0003	cytoskeleton/motility
BC034535	KRT6B	0.005	0.0043	cytoskeleton/motility
M19156	KRT10	0.018	0.001	cytoskeleton/motility
AJ551176	SDC1	0.039	0.0038	cytoskeleton/motility
NM_006478	GAS2L1	0.22	0.0016	cytoskeleton/motility
M34225	KRT8	0.26	0.0029	cytoskeleton/motility
NM_005886	KATNB1	0.27	0.0011	cytoskeleton/motility
AK024835	CNN2	0.47	0.003	cytoskeleton/motility
NM_006350	FST	0.11	0.00001	extracellular matrix
AF177941	COLSA3	0.14	0.00001	extracellular matrix
L22548	COL18A1	0.49	0.0011	extracellular matrix
M58051	FGFR3	0.007	0.0039	growth factor receptor
NM_004887	CXCL14	0.009	0.0014	chemokine
AF289090	BMP7	0.13	0.002	cytokine
K03222	TGFA	0.2	0.0048	growth factor
M31682	INHBB	0.20	0.00001	cytokine
NM_004750	CRLF1	0.26	0.0003	cytokine binding
NM_002514	NOV (CCN3)	0.28	0.0009	growth factor
NM_000685	AGTR1	0.30	0.005	growth factor receptor
D16431	HDGF	0.42	0.0046	creatine kinase
L36719	MAP2K3	0.22	0.0048	protein kinase activity
AJ290975	ITPKC	0.28	0.0036	protein kinase activity
NM_001569	IRAK1	0.33	0.0001	protein kinase activity
AB025285	ERBB2	0.45	0.0003	protein kinase
AF029082	SFN	0.001	0.0028	signal transduction
AB065865	HM74	0.04	0.0047	signal transduction
AA021034	LTB4R	0.06	0.0006	signal transduction
NM_004445	EPHB6	0.12	0.0038	signal transduction
AF025304	EPHB2	0.17	0.0021	signal transduction
AB026663	MC1R	0.17	0.0046	signal transduction
AF035442	VAV3	0.17	0.004	signal transduction
NM_014030	GIT1	0.21	0.0025	signal transduction
AB011152	CENTD1	0.21	0.0003	signal transduction
AK095244	CYB561	0.23	0.0001	signal transduction
AF106858	GPR56	0.23	0.0002	signal transduction
AF231024	CELSR1	0.23	0.0006	signal transduction
AF234887	CELSR2	0.24	0.0003	signal transduction
NM_007197	FZD10	0.25	0.0009	signal transduction
NM_014349	APOL3	0.25	0.002	signal transduction
NM_004039	ANXA2	0.27	0.0044	signal transduction
AI285986	THBD	0.29	0.0004	signal transduction
M57730	EFNA1	0.31	0.0032	signal transduction
NM_002118	HLA-DMB	0.33	0.0008	signal transduction
AF427491	TUBB4	0.36	0.001	signal transduction
NM_005279	GPR1	0.40	0.0033	signal transduction
X60592	TNFRSF5	0.40	0.0032	signal transduction
BC052968	EPHB3	0.42	0.0001	signal transduction
M64749	CMKOR1	0.46	0.0014	signal transduction
M21188	IDE	0.46	0.0031	signal transduction
AB018325	CENTD2	0.47	0.0004	signal transduction
AK054968	ITGB5	0.49	0.0005	signal transduction
NM_001730	KLF5	0.04	0.0021	transcription factor
NM_004350	RUNX3	0.08	0.0001	transcription factor
U34070	CEBPA	0.11	0.0005	transcription factor
AF062649	PTTG1	0.15	0.0039	transcription factor
NM_004235	KLF4	0.20	0.0005	transcription factor
X52773	RXRA	0.20	0.0011	transcription factor
AF202118	HOXD1	0.21	0.0006	transcription factor
NM_000376	VDR	0.21	0.0001	transcription factor
NM_006548	IMP-2	0.26	0.0031	transcription factor
NM_007315	STAT1	0.32	0.00001	transcription factor
NM_004430	EGR3	0.34	0.002	transcription factor
NM_003644	GAS7	0.36	0.0033	transcription factor
NM_005900	MADH1	0.48	0.0028	transcription factor
X14454	IRF1	0.49	0.0013	transcription factor
AF067572	STAT6	0.49	0.0001	transcription factor
NM_005596	NFIB	0.49	0.0041	transcription factor
AB002282	EDF1	0.40	0.0002	transcription coactivator
AK075393	CTSB	0.50	0.0016	protease activity
AB021227	MMP24	0.29	0.0001	protease activity
AB007774	CSTA	0.02	0.0018	cysteine protease inhibitor
AF143883	ALOX12	0.06	0.0016	catalytic activity
AF440204	PTGS1	0.08	0.00001	catalytic activity
NM_000777	CYP3A5	0.14	0.0041	catalytic activity
NM_016593	CYP39A1	0.21	0.0027	catalytic activity
BC001491	HMOX1	0.23	0.0028	catalytic activity
BC020734	PGDS	0.26	0.00001	catalytic activity
AL133324	GSS	0.39	0.002	catalytic activity
AF055027	CARM1	0.41	0.00001	catalytic activity
NM_001630	ANXA8	0.01	0.0006	calcium ion binding
AB011542	EGFL5	0.43	0.0001	calcium ion binding
NM_005979	S100A13	0.31	0.001	calcium ion binding
NM_020672	S100A14	0.02	0.0005	calcium ion binding
NM_005978	S100A2	0.003	0.005	calcium ion binding
BC012610	HF1	0.22	0.00001	complement activation
AF052692	GJB3	0.03	0.0001	connexon channel activity
M12529	APOE	0.21	0.0001	metabolism
NM_004925	AQP3	0.01	0.0003	transporter activity

Partial list of differentially expressed genes identified in leiomyomas (African Americans and Caucasians) as compared to keloid/incisional scars as shown in Fig. 1. The genes were selected based on p ranking of p ≤ 0.005 and 2-fold cutoff change selection (F. Change) as described in materials and methods. Table 2 displays the under-expressed genes in leiomyomas as compared to keloid/incisional scars.

The analysis based on inclusion of leiomyomas as two independent cohorts (3 A. American and 3 Caucasians) resulted in identification of a limited number of differentially expressed genes as compared to keloids (N = 2)/incisional scars (N = 2). Because both keloids were from A. American patients we excluded one of the incisional scar from a Caucasian patient from the analysis and lowered the statistical stringency to P < 0.01 which resulted in identified 424 differentially expressed genes in A. American leiomyomas as compared to keloids/scars. Similar analysis resulted in identified 393 differentially expressed genes in Caucasian leiomyomas as compared to keloids/scars (all from A. Americans). Of these genes 64 and 32 genes, respectively differed by at least 2 fold in leiomyomas of AA and Caucasians, compared to keloids/incisional scars (Table 3).

**Table 3:** Differentially expressed genes in leiomyomas compared to keloids/incesional scars

**Gene Bank**	**Symbol**	**F. Change** **LAA:Scar**	**F. Change** **LC:Scar**	**P value**	**Function**
NM_006198	PCP4	68.14	6.66	0.0017	system development
S67238	MYOSIN	62.78	36.69	0.0034	cytoskeleton/motility
NM_004342	Cald1	21.43	9.32	0.0047	cytoskeleton/motility
NM_013437	LRP12	20.6	6.82	0.0053	cellular process
AC004010	AMIGO2	19.07	10.61	0.0021	cell adhesion
AF040254	OCX	18.71	5.39	0.0099	signal transduction
NM_015385	SORBS1	17.44	9.26	0.0003	cytoskeleton/motility
NM_012278	ITGB1BP2	17.42	9.9	0.0018	signal transduction
NM_006101	KNTC2	17.33	5.23	0.0022	transcription factor
NM_001845	COL4A1	16.08	5.94	0.0029	cytoskeleton/motility
AF104857	CDC42EP3	16.08	3.78	0.0002	cytoskeleton/motility
AW188131	DDX17	15.65	9.11	0.0005	translation factor
NM_001057	TACR2	15.6	4.51	0.0062	signal transduction
AI375002	ZNF447	14.55	8.04	0.0061	transcription factor
NM_014890	DOC1	14.35	5.19	0.0002	proteolysis
NM_001784	CD97	13.16	6.35	0.00004	signal transduction
BF111821	WSB1	12.34	7.36	0.0024	signal transduction
AW152664	PNN	12.19	8.26	0.003	transcription factor
NM_002380	MATN2	11.86	5.62	0.0011	extracellular matrix
NM_007362	NCBP2	11.38	8.04	0.0034	RNA processing
AK023406	Macf1	8.8	4.77	0.0041	ECM signaling
AF095192	BAG2	8.01	4.34	0.0018	apoptosis
NM_004196	CDKL1	7.91	2.83	0.0017	cell cycle
BF512200	MBNL2	7.58	3.01	0.0014	muscle differentiaon
AW043713	Sulfl	6.9	0.78	0.0039	hydrolase activity
NM_004781	VAMP3	6.76	3.02	0.0016	trafficking
AI149535	STAT5B	5.62	3.94	0.0043	transcription factor
NM_016277	RAB23	5.61	2.68	0.0055	signal transduction
AI582238	TRA1	5.13	3.46	0.0042	calcium ion binding
NM_005722	ACTR2	4.04	2.49	0.0001	cytoskeleton/motility
AF016005	RERE	4.02	2.87	0.008	transcription factor
AL046979	TNS1	3.65	2.14	0.0047	signal transduction
NM_005757	MBNL2	3.57	0.84	0.0049	muscle development
AJ133768	LDB3	3.3	1.53	0.0056	cytoskeleton/motility
AI650819	CUL4B	3.04	1.59	0.0045	metabolism
AL031602	MT1K	0.61	0.33	0.0086	cadmium ion binding
U85658	TFAP2C	0.27	0.14	0.0083	transcription factor
NM_003790	TNFRSF25	0.19	0.11	0.007	apoptosis
BC002495	BAIAP2	0.18	0.11	0.0003	signal transduction
AV691491	TMEM30B	0.13	0.09	0.0093	cell cycle control
AI889941	COL4A6	10.4	30.21	0.007	extracellular matrix
AW451711	PBX1	14.44	18.14	0.0001	transcription factor
NM_014668	GREB1	7.18	15.94	0.0089	
NM_004619	TRAF5	6.47	11.46	0.0091	signal transduction
NM_005418	ST5	5.83	8.1	0.0044	signal transduction
BC002811	SUMO2	0.47	0.83	0.0035	protein binding
AV700891	ETS2	0.28	0.54	0.0082	transcription factor
AB042557	PDE4DIP	0.2	0.39	0.0019	signaling
NM_014485	PGDS	0.17	0.31	0.0027	catalytic activity
AI984221	COL5A3	0.08	0.17	0.0011	extracellular matrix
NM_006823	PKIA	0.08	0.17	0.0034	Kinase regulator
AU144284	IRF6	0.04	0.15	0.0026	transcription factor
NM_000962	PTGS1	0.06	0.11	0.0046	catalytic activity
NM_022898	BCL11B	0.05	0.09	0.0099	transcription factor
NM_001982	ERBB3	0.02	0.06	0.0066	signal transduction
NM_002705	PPL	0.005	0.031	0.0073	hydrolase activity
NM_001630	ANXA8	0.006	0.02	0.0079	calcium ion binding
N74607	AQP3	0.006	0.02	0.0098	transporter activity
NM_000142	FGFR3	0.007	0.009	0.01	Growth factor
Receptor					

**Figure 2:**
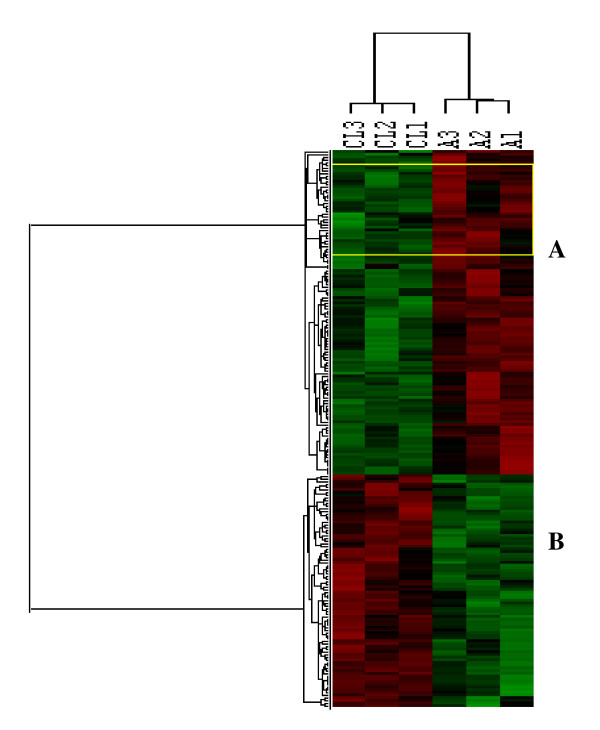
Cluster analysis of 206 differentially expressed genes in leiomyomas from Caucasians (CL1, CL2, and CL3) and peritoneal adhesions (A1, A2, A3) using Affymetrix U95 array. The genes were selected based on supervised and unsupervised assessment and p ranking at P < 0.01 followed by 2-fold cutoff change selection. The genes represented by rows were clustered according to their similarities in expression patterns for each tissue and identified as A and B.

Partial list of differentially expressed genes from several functional categories in leiomyomas from African Americans and Caucasians as compared to keloids/incesional scars as shown in Fig. 2. The genes were selected based on p ranking of p ≤ 0.01 and following 2-fold cutoff change

We also utilized the gene expression values obtained in our previous microarray studies in leiomyomas[11] and peritoneal adhesions (unpublished results) for comparative analysis. Because these results were generated using Affymetrix U95A GeneChips, due to cross-platform comparability with U133A the combined data from both platforms were subjected to additional analysis as described in the materials and methods. The analysis based on p < 0.005 and 2-fold change cutoff identified 1801 genes as over-expressed and 45 under-expressed in leiomyomas as compared to keloids/incisional scars and peritoneal adhesions (considered as one cohort during analysis). Of these, 85 genes were differentially expressed in leiomyomas as compared to peritoneal adhesions (Fig. 2), however exclusion of U133A data from the analysis resulted in identification of a higher number differentially expressed genes. The gene expression profiles in these tissues were comparatively analyzed with their corresponding normal tissues, myometrium, skin and peritoneum, and as expected they displayed distinct patterns (data not shown). The analysis confirmed the effect of cross-platform on gene expression profiling when comparing results of different studies (See Nature Bio-technology, Sept 2006 for several reviews).

### Realtime PCR of gene expression

**Figure 3:**
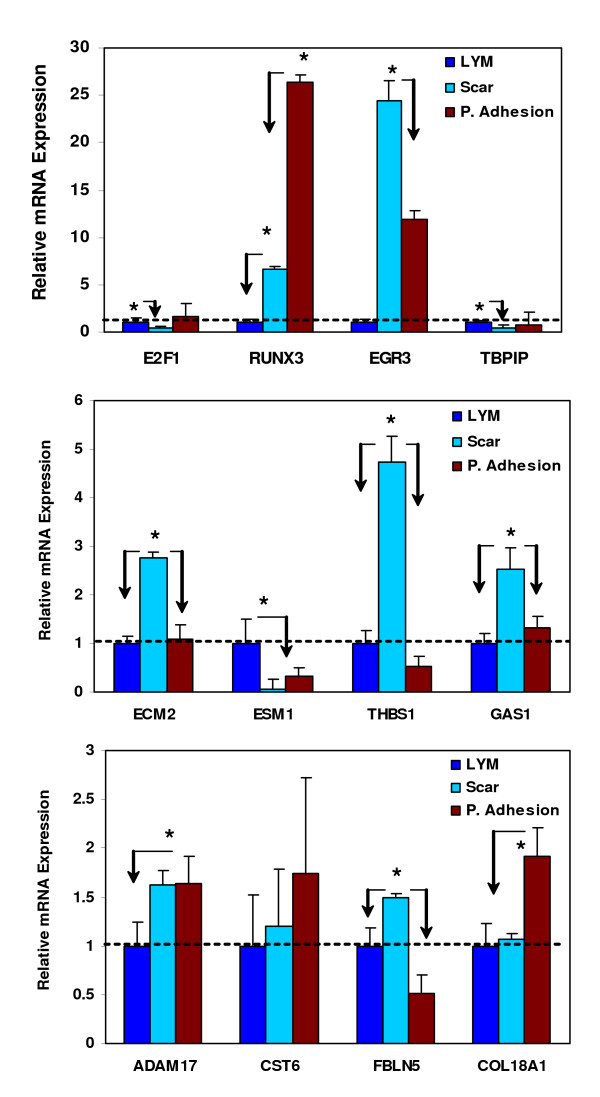
The bar graphs show the relative mean expression levels of 12 genes (E2F1, RUNX3, EGR3, TBPIP, ECM-2 ESM1, THBS1, GAS1, ADAM17, CST6, FBLN5, and COL18A1) in leiomyomas (LYM), keloids/incisional scars (Scar) and peritoneal adhesions (P. Adhesion) using realtime PCR and LDA as described in materials and methods section. Values on the y-axis represent an arbitrary unit derived from the mean expression level of these genes in each tissue with their mean expression values in leiomyomas set at 1 independently for each gene prior to normalization against their expression levels in myometrium form a Caucasian serving as control. The asterisks * indicate statistical difference between the expression of these genes with arrows pointing the difference between each group. A probability level of P < 0.05 was considered significant.

Gene ontology assessment and division into functional categories indicated that a majority of the differentially expressed genes identified in these cohorts serve as regulator of transcription, cell cycle and apoptosis, extracellular matrix turnover, adhesion molecules, signal transduction and transcription factors (Tables 1, 2 and 3). Since the expression of E2F1, RUNX3, EGR3, TBPIP, ECM-2, ESM1, THBS1, GAS1, ADAM17, CST6, FBLN5, and COL18A1 was evaluated in leiomyomas using LDA-based realtime PCR as described in the accompanying manuscript [17] we used the same approach and compared their expression in keloids, incisional scars and peritoneal adhesions. The level of expression of these 12 genes displayed significant variations among these tissues with some overlapping patterns with the microarray results. By setting the mean expression value of each gene independently as 1 in leiomyomas compared with their mean expression in keloids/incisional scars (scar) and adhesions, the results indicated that the expression of E2F1, TBPIP and ESM1 was elevated in leiomyoma as compared to keloids/incisional scars and adhesions (Fig. 3, P < 0.05). In contrast, the expression of EGR3, ECM2, THBS1, GAS1 and FBLN5 in scars and RUNX3 and COL18 expression in peritoneal adhesions was higher as compared to leiomyomas (Fig. 3).

## Discussion

Using a large-scale gene expression profiling approach we compared leiomyomas with keloids, incisional cars and peritoneal adhesions and found that their molecular environments consist of a combination of both tissue-specific and commonly expressed genes. The tissue-specific gene expression between leiomyomas and keloids was not reflected based on the presence/absence of unique genes, but rather occurred at the level of expression of a selective number of differentially expressed genes. As such an elevated level of expression of a number of muscle cell-specific genes in leiomyomas and fibroblast-specific genes in keloids reflected the specific cellular make up of these tissues. In addition, specific expression of estrogen receptor (ER) in leiomyomas with limited expression in keloids and incesional scar tissues re-enforced the importance of ovarian steroids in leiomyomas growth. Collectively the results suggest that the molecular environments that govern the characteristic of these fibrotic tissues, at least at genomic levels, are relatively similar and involved specific set of genes represented by 3 to 12% of the genes on the array. This observation also suggests that differential expression of a limited number of these genes with unique biological functions may regulate the processes that results in establishment and progression of leiomyoma, keloids, incisional scars, and possibly other fibrotic disorders, despite differences in the nature of their development and growth.

We recognize that the stage of the menstrual cycle and to a limited extend the size of leiomyomas, as well as the period since keloids, incisional scars and peritoneal adhesions were first formed, reflecting the stage of wound healing, influences the outcome of their gene expression. Although leiomyomas used in our study were similar in size and from the same phase of the menstrual cycle, the stage of keloids and scars tissues was unknown. As such the study results represent their gene expression at the time of collection. We also recognize that small sample size limited our ability to analyze the data based on ethnicity, because of more frequent development of leiomyomas and keloids in African Americans. However, it is worth mentioning that comparing leiomyomas with keloids from this ethnic group showed a limited difference in their gene expression profile, or when compared with leiomyomas from Caucasians, suggesting the existence of a comparable environment in leiomyomas and keloids. Further comparison of leiomyomas' gene expression with peritoneal adhesions (Affymetrix U95A subjected to cross-platform comparability analysis) also identified a low number of differentially expressed genes (85 genes) in these tissues, although analysis based only on U95A arrays identified higher numbers. The results indicate that the molecular environment of leiomyomas may be more comparable to peritoneal adhesions as compared to keloids/incisional scars at least at late stage of their wound healing development. Possibly the size of leiomyomas (larger size often undergoing degeneration at the center), and the stage of keloids, incesional scars and adhesions formation following tissue injury influencing their gene expression profiles would produce different results from our study and their evaluation would enhance our understanding of molecular conditions that lead to tissue fibrosis at these and other sites [18-21].

A majority of the genes identified in leiomyomas, keloid, incisional scars and adhesions function as regulators of cell survival (cell cycle and apoptosis), cell and tissue structure (ECM, adhesion molecules and cytoskeleton), tissue turnover, inflammatory mediators, signal transduction and transcription and metabolism. Consistent with the importance of ECM, cytoskeleton, adhesion molecules and proteases in tissue fibrosis we identified the expression of many of genes in these categories some with 5 to 60 fold increase in their expression. Elevated expression of DES, MYH11, MYL9 and SMTN in leiomyomas and several KRTs in keloids and scars reflects the cellular composition of these tissues. Additionally, PALLD has been considered to serve as a novel marker of myofibroblast conversion and is regulated by profibrotic cytokine such as TGF-β [22,23]. SM22, which is overexpressed in keloids[24], promotes ECM accumulation through inhibition of MMP-9 expression [25]. The expression of many components of ECM including collagens, decorin, versican, fibromodulin, intergrins, extracellular matrix protein 1 (ECM-1), syndecan and ESM-1 has been identified in leiomyomas [11,17,26] as well as dermal wounds during healing, scars and keloids (for review see [27-32]).

We validated the expression of ECM-2, ESM1, THBS1, FBLN5 and COL18A1 in keloids, incisional scars and adhesions and the analysis indicated an elevated expression of ECM2, THBS1 and FBLN5 in keloid/incisional scars and COL18 in peritoneal adhesions as compared to leiomyomas[17]. Although the biological significance of these gene products and changes in their expression in leiomyomas, keloids and adhesions remains to be established, the product of a specific number of these genes such as ECMs, THBS1, FBLNs, MMPs and ADAMs play a critical role in various aspect of wound healing and tissue fibrosis [27-32]. A number of MMPs were equally expressed in leiomyomas, keloids and peritoneal adhesions with the exception of lower MMP-14, MMP-24 and MMP-28 expression in leiomyomas, suggesting that these tissues are potential target of their proteolytic actions. The biological importance of lower expression of these MMPs in leiomyoma is unknown; however unlike most MMPs that are secreted as inactive proenzymes and require activation, MMP-11 and MMP-28 are secreted in active forms. In keratinocytes, MMP-28 is expressed in response to injury and detected in the conditioned media of hypertrophic scars, but not normotrophic scars [33]. A lower expression of MMP-28 and elevated expression of TIMP-3 in leiomyomas compared to keloids imply a lower matrix turnover with an increase angiogenic and pro-apoptotic activities that has been associated with TIMP-3 [34,35].

We identified an overexpression of a higher number of apoptotic-related genes in keloids and incisional scars as compared to leiomyomas, suggesting an increased rate of cellular turnover. Because apoptotic and non-apoptotic cell death is considered to increase local inflammatory reaction and a key step in tissue fibrosis, a number of genes functionally categorized as proinflammatory and pro-fibrotic mediators were identified in these tissues. Noticeable among these genes were TGF-β, IL-1, IL-6, IL-11, IL-13, IL-17, IL-22 and IL-27 and chemokines CCL-2 to 5, CX3-CL1, CXCL-1, CXCL-12 and CXCL-14 and their receptors. Elevated expression of PDGF-C, VEGF and FGF2 in leiomyomas as compared to keloids and adhesions imply an additional role for these angiogenic factors in pathogenesis of leiomyomas. While the expression of TGF-β was equally elevated in leiomyomas, keloids, incisional scars and peritoneal adhesion as compared to their normal tissues reinforcing the importance of TGF-β as principle mediator of tissue fibrosis [30]. Although profibrotic action of TGF-β is reported to involve the induction of CTGF, a member of PDGF family with mitogen action for myofibroblasts [36], it is expressed at lower levels in leiomyomas as compared to myometrium [26,37,38]. However, leiomyomas of African Americans expressed a 3.3 fold higher levels of CTGF as compared to Caucasians, and 12.6 and 4.3 fold higher as compared to keloids and incisional scars, respectively. Although the biological significance of these differences needs further investigation, altered expression of many of these genes as compared to their normal tissues counterpart also imply their potential role in various cellular processes that results in tissue fibrosis.

The genes encoding signal transduction and transcription factors represented the largest functional category in leiomyomas and scar tissues. They included several genes such as NR2F1, PNN, Smad4, Smad5, STAT5B, JUN, TGIF2, and ATF1 that were over-expressed while RUNX3, STAT1, STAT6, EGR3, GAS7, Smad1, and EDF1 were underexpressed in leiomyomas as compared to keloid/incisional scars. We validated the expression of E2F1, RUNX3, EGR3 and TBPIP in leiomyomas [17], keloids, incisional scars and peritoneal adhesions showing a good correlation with microarray data Since activation of these signal transduction pathways and transcription factors regulate the expression of large number of genes with diverse functional activities their altered expression in these tissues could have a considerably more important role in tissue fibrosis than previously considered. Preferential phosphorylation of many of these transcription factors such as Jun, Stats, Smads, Runx and EGRs leads to regulation of target genes involved in cell growth and apoptosis, inflammation, angiogenesis and tissue turnover with central roles in tissue fibrosis [11,17,39-42]

In conclusion, the gene expression profiling involving leiomyomas and their comparison with keloids, incisional scars and peritoneal adhesion indicated that a combination of tissue-specific and common genes differentiate their molecular environments. The tissue-specific differences were not based on the presence/absence of unique genes, but rather the level of expression of selective number of genes accounting for 3 to 12% of the genes on the array. Although the nature of leiomyomas' development and growth is vastly different from these fibrotic tissues, we speculate that the outcome of their tissue characteristics is influenced by the products of genes regulating cell growth and apoptosis, inflammation, angiogenesis and tissue turnover, and may also be under different tissue-specific regulatory control.

## Competing interests

The author(s) declare that they have no competing interests.

## Authors' contributions

XL, QP and NC participated in all aspect of the experimental design and writing of the work presented here. The final microarray gene chips were performed at Interdisciplinary Center for Biotechnology Research at the University of Florida. The analysis of microarray gene expression profiles between the gene chips U95 and 133a was carried out by LL and gene expression analysis and realtime PCR was performed by XL and QP. All the authors read and approved the final manuscript.
